# Evaluating National Imaging Guidelines for Rotator Cuff Assessment After Shoulder Dislocation in Adults Aged 40–60 Years

**DOI:** 10.7759/cureus.96840

**Published:** 2025-11-14

**Authors:** Max Moss, Matthieu Durand-Hill, Saneth Sellahewa, Dylan Griffiths, Sanjeeve Sabharwal

**Affiliations:** 1 Trauma and Orthopaedics, Royal Bolton Hospital, Farnworth, GBR; 2 Trauma and Orthopaedics, London North West University Healthcare NHS Trust, Harrow, GBR; 3 Trauma and Orthopaedics, Imperial College London, London, GBR

**Keywords:** first-time shoulder dislocation, mri shoulder, recurrent shoulder dislocation, rotator cuff tears, ultrasound (u/s)

## Abstract

Introduction: Rotator cuff tears are a recognised complication of shoulder dislocation. The British Elbow and Shoulder Society (BESS) recommends further imaging via ultrasound (US) or MRI to assess for clinically significant cuff tears in patients aged 40-60 years. This study aims to produce supplementary evidence by assessing cuff tear incidence to help assess the utility of imaging and its impact on resources.

Methods: This was a retrospective observational study to assess imaging and management of patients aged 40-60 years presenting with shoulder dislocation. Data was collected for three years, 2021-2024. The number of patients undergoing MRI/US and the prevalence of cuff tear were collected. Patients were analysed for conservative or operative management.

Results: A total of 148 patients were collected (mean age 49.5 years, SD 6.5). Of these, 68 patients (45.9%) had further cross-sectional imaging via MRI/US, and of the 68 patients, 30 (44.1%) had radiologically reported rotator cuff tears. A total of 116 patients (78.4%) were followed up after dislocation. One patient proceeded to surgery for rotator cuff repair following diagnosis.

Conclusion: This study highlights the high rate of imaging-detected rotator cuff tears following shoulder dislocation in patients aged 40-60 years, yet a very low rate of surgical intervention. A more symptom-guided imaging strategy may improve clinical relevance and reduce unnecessary use of advanced imaging resources.

## Introduction

Rotator cuff tears are a recognised complication of shoulder dislocation, with incidence rising with age. Prior studies suggest radiologically confirmed tears in approximately 40% of patients aged 40-55 years, over 70% of those aged 55-70, and nearly universal occurrence beyond 70 years [1-3].

The British Elbow and Shoulder Society (BESS) has therefore recommended cross-sectional imaging via ultrasound (US) or MRI in patients aged 40-60 years, citing elevated risk of rotator cuff pathology [4]. However, these recommendations are supported by limited evidence, most notably by a single study of 87 patients by Simank et al. [1]. Despite widespread adoption of BESS guidance, it remains uncertain whether routine imaging in this age group meaningfully influences patient management. The increasing reliance on imaging in modern clinical practice may stem from a desire to identify pathology early to prevent complications or guide management. However, the early use of routine imaging in this age bracket, especially in those without symptoms, remains a controversial topic.

Identifying a cuff tear radiologically may not necessarily alter treatment decisions, particularly given the high proportion of patients managed conservatively with physiotherapy rather than with surgery. Previous studies have highlighted a disparity between the imaging findings in these patients and the clinical outcomes in this cohort. A significant portion of patients will have a degree of cuff pathology as mentioned earlier, yet few will proceed to surgical intervention [5]. Additionally, cuff abnormalities may be detected on advanced imaging in this age cohort, but often will not correlate with ongoing symptoms or functional limitations [6]; this indicates that a more selective rather than universal imaging approach may be more appropriate if utilised alongside patient symptoms and dysfunction on clinical review.

Routine imaging also carries the risk of overdiagnosing and treating patients who otherwise have low symptomatic or functional deficits, and a more targeted imaging approach may prove beneficial for healthcare trusts and patients with regard to economics and patient experience. Earlier studies have shown that despite accurate detection of additional cuff abnormalities, the findings minimally alter patient management [7]. Moreover, it has been shown that surgical decision-making is primarily driven by patient history and examination, with specialist imaging playing a minor role [8]. The economic implications of such routine imaging protocols alongside poor resource utilisation and delays in care can bring about debate regarding the necessity of universal imaging in this age cohort following shoulder dislocation, and that further specialist review could be a more effective strategy.

The aims of this study were to assess real-world adherence to BESS guidance and to quantify the incidence of rotator cuff tears and explore whether identification of a tear had clinical relevance in terms of subsequent treatment decisions, thereby assessing the clinical utility and resource implications of current BESS recommendations. This would help generate supplementary evidence to assess the practical utility and resource implications of current national imaging guidance. 

## Materials and methods

Study design and setting

This was a retrospective cohort study of patients aged 40-60 years who presented with a primary or recurrent shoulder dislocation at the Imperial College Healthcare NHS Trust, London, United Kingdom, over a three-year period. Based on BESS national guidance and historical poor local compliance, a pathway was introduced prior to the data collection period to create a route for imaging these acute shoulder dislocation patients either through a virtual or face-to-face fracture clinic. This was a single-centre service evaluation registered with the local audit department; formal ethical approval was not required in accordance with internal governance protocols.

Eligibility criteria

Inclusion criteria consisted of patients aged between 40 and 60 years of age with a confirmed shoulder dislocation who had been referred to the virtual or face-to-face fracture clinic pathway for imaging. Data was collected from patients who presented to the Trust between August 2021 to May 2024. Patients were excluded if there was no radiological evidence or written documentation of acute shoulder dislocations.

Data collection

The patient database was developed by IT specialists in the local audit department based on patients referred to the local pathway for imaging following shoulder dislocation in this age cohort. Data on patient demographics (age, sex), comorbidities (alcohol excess, steroid use, rheumatoid arthritis, diabetic status), and clinical management decisions were collected from electronic health records (Oracle Health (formerly Cerner), Kansas City, Missouri, United Kingdom). This included documentation in inpatient notes and outpatient follow-up appointment letters. Patient management outcome details included those on discharge, conservative, or surgical strategies. Picture Archiving and Communication System (PACS) radiology systems enabled the collection of data regarding date and laterality of dislocation, the type of further imaging performed (US/MRI), and the consequent radiological report on the presence of rotator cuff tear.

Data analysis

Data was collected and analysed descriptively using Microsoft Excel (Version 16.77.1; Microsoft Corporation, Redmond, Washington, United States). Mean and standard deviation (SD) were calculated for continuous data. Prevalence of rotator cuff tears was calculated as the percentage of patients who had a radiologically diagnosed rotator cuff tear in those who underwent US/MRI investigations following shoulder dislocation. Incidence of surgery was calculated as a proportion of those who underwent rotator cuff repair following radiological diagnosis of cuff tear.

## Results

A total of 148 patients between 40 and 60 years of age (mean 49.5±6.5 years) presented with shoulder dislocations and were included in the study. Of these, 102 (68.9%) patients were male, 42 (28.4%) were female, and data on sex were unavailable for four (2.8%). Seventeen (11.4%) had a history of alcohol excess, one (0.7%) reported steroid use, one (0.7%) had rheumatoid arthritis, and eight (5.4%) were diabetic. Of 148 patients, only 68 underwent MRI/US, of whom 44% had a rotator cuff tear. Yet only one patient proceeded to surgical cuff repair. The breakdown of patient demographics and results can be seen in Table 1.

**Table 1:** Patient demographics and clinical findings (N=148)

Parameter	Frequency (Percentage)
Gender	
Male	102 (68.9%)
Female	42 (28.4%)
Unavailable/other	4 (2.7%)
Comorbidities	
Alcohol excess	17 (11.4%)
Diabetes mellitus	8 (5.4%)
Steroid use	1 (0.7%)
Rheumatoid arthritis	1 (0.7%)
Evidence of Dislocation	
X-Ray evidence	132 (89.2%)
CT evidence	4 (2.7%)
Non-UK hospitals	3 (2.0%)
Documented self-reduction	8 (5.4%)
Reduction in clinical diagnosis	1 (0.7)
Imaging (MRI/Ultrasound)	
Underwent MRI/Ultrasound	68 (45.9%)
Incidence of rotator cuff tear	30 (44.1% of 68)
Required rotator cuff repair	1 (3.3% of 30)

**Figure 1:**
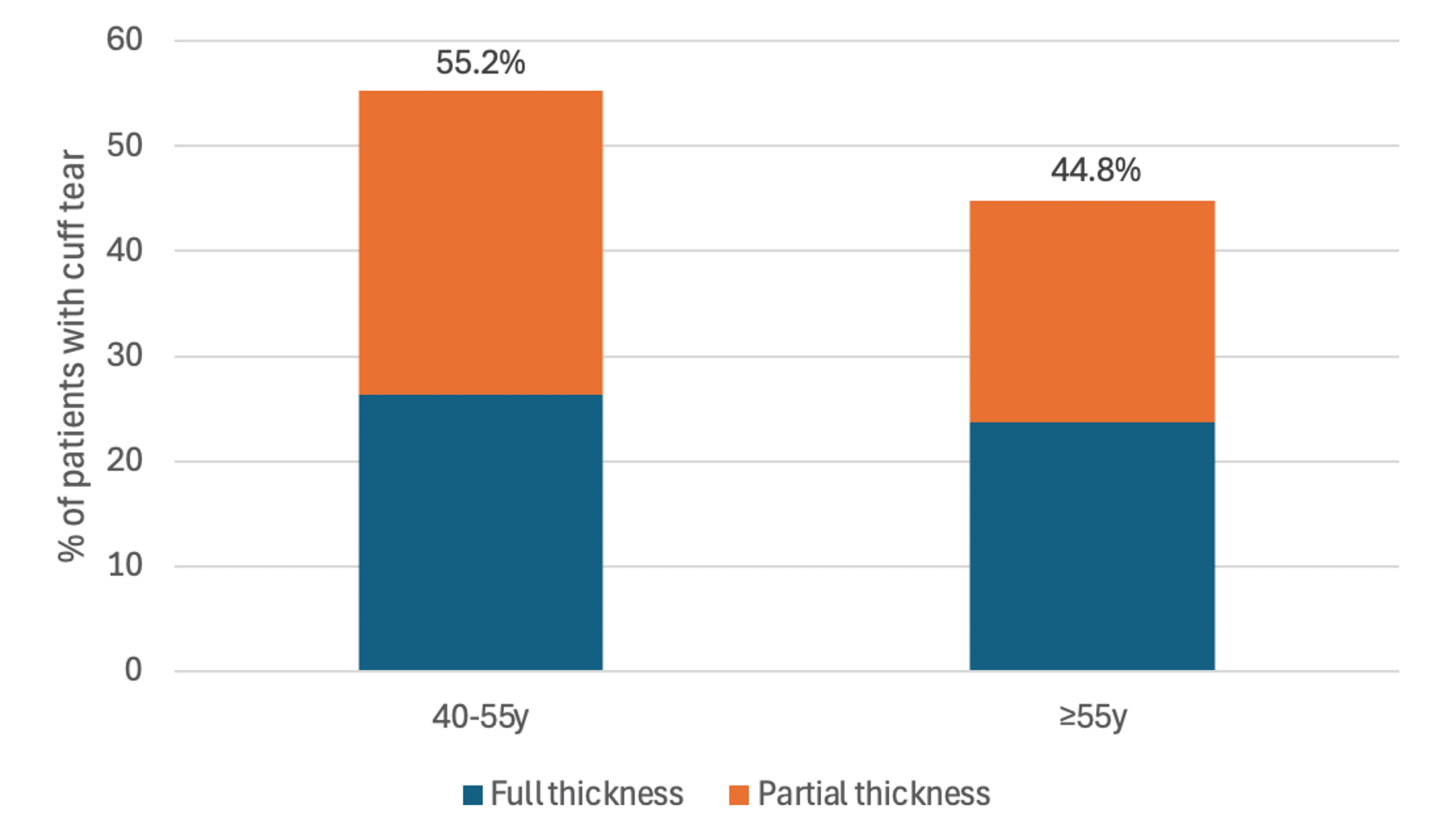
Incidence and type of rotator cuff tears by age (N=30) Data presented as percentages

A total of 132 patients had plain radiographic evidence of dislocation, and four patients had CT evidence of shoulder dislocation. Three patients had imaging confirmation of dislocation in non-UK hospitals. Eight (4.8%) patients had documented self-reduction of known/recurrent dislocation, and one (0.6%) patient was reduced in A&E following clinical diagnosis. Sixty-eight patients underwent investigation with MRI or US. Of these, 30 (44.1%) patients had rotator cuff tears as stated on radiology reports. A breakdown of the incidence of rotator cuff tears by age is shown in Figure 1. A full breakdown of rotator cuff tears is presented in Table 2.

**Table 2:** Distribution of rotator cuff tears according to type and muscle

Muscle	Partial-thickness tear, n	Full-thickness tear, n	Total, n
Supraspinatus	13	14	27
Infraspinatus	3	2	5
Subscapularis	5	2	7
Teres Minor	0	0	0

One patient from the sample underwent surgery for rotator cuff repair. Two patients underwent anterior stabilisation for recurrent dislocation (neither had a radiological diagnosis of rotator cuff tear). Three patients required urgent open reduction internal fixation for associated fractures, with no further investigation for rotator cuff pathology. Two further patients were listed for surgery but found to be medically unfit for the procedure.

A total of 116 (78.4%) patients were followed up following dislocation. Of these, 74 (50%) patients were reviewed face-to-face and discharged from the clinic and physiotherapy following functional and symptomatic recovery, and 10 (6.8%) patients were reviewed in the clinic and required hydrodistension therapy prior to discharge from the clinic and physiotherapy. Seven (4.7%) patients had ongoing physiotherapy following discharge from the clinic. Seventeen (11.5%) patients were contacted via telephone following virtual fracture clinic review and discharged with safety netting advice. The eight (5.4%) remaining patients were the patients followed up for surgery (performed or cancelled), stabilisation, or open reduction and internal fixation (ORIF). A total of 27 (21.6%) patients either did not attend clinic appointments, were repatriated to local hospitals, or opted for private care following dislocation.

Of the 68 patients who underwent MRI or US following dislocation, 45 (66.1%) had their US/MRI following the first clinical review, and 23 (33.9%) had this imaging prior to the first face-to-face clinical review.

## Discussion

Our study highlights a disconnect between the high prevalence of imaging-detected cuff tears and the very low rate of surgical repair. This raises questions about the BESS guidance for routine imaging in all patients aged 40-60 years, after shoulder dislocation.

For the patients who underwent MRI/US following dislocation, the incidence of rotator cuff pathology was high (44.1%). However, this is not a true reflection of rotator cuff tear incidence within this cohort of 148 patients, but only of the patients who had further imaging following dislocation. Interestingly, despite the presence of well-established cuff pathology within the imaged and followed-up cohort, only one patient proceeded to surgical intervention for cuff repair. Additionally, analysis of patient follow-up revealed a large proportion underwent serial clinical reviews following dislocation to determine management. Ongoing imaging and surgical decisions were dependent on these consultations, with two-thirds of patients having MRI/US after consultation and nearly 75% of patients being discharged with physiotherapy. These findings prompt a critical evaluation of the necessity for routine advanced imaging in all patients aged 40-60 following shoulder dislocation.

Currently, BESS recommends that patients >40 years can mobilise as pain allows following shoulder dislocation and warrant further imaging to assess for shoulder instability in the form of MRI/US. The guideline is non-specific relating to imaging due to no statistical difference in diagnostics between the two modalities [9-13], with MRI being the more expensive to perform [14,15]. BESS does not include specific recommendations relating to follow-up with these patients surrounding inpatient reviews prior to requesting further imaging. In the context of a resource-constrained healthcare system like the NHS, it’s essential to balance the clinical utility of such imaging against its cost and logistical burden. Results from this study suggest a more selective, symptom-driven approach to imaging may be both clinically appropriate and economically prudent.

Of the patients who had further cross-sectional imaging following shoulder dislocation, 41.4% were diagnosed with a rotator cuff tear. Similar to our data, Simank et al. recorded a 41% incidence in patients aged 40-55 [1]. In another study, 38% of patients were diagnosed with a cuff tear following shoulder dislocation [16], with further replicated work showing a prevalence of 31% in a similar age cohort [2]. Results from the subset of patients imaged within our cohort are comparable to previous data on rotator cuff tears post dislocation and provide support for imaging patients following dislocation in this age group as per BESS. But as stated earlier, this incidence is not a true reflection of the entire sample. It is important to consider the need to routinely image all patients post shoulder dislocation in the 40-60 age cohort, rather than opting for a more selective and symptom-driven approach in patient selection. The analysis of the management history for patients in our study shows a high number of patients can be managed conservatively with physiotherapy, with multiple clinical reviews to assess functional and symptomatic recovery with or without radiological diagnosis of rotator cuff tear. Furthermore, adjuncts such as evolving symptomatic history and specific clinical tests could be used in conjunction with BESS imaging guidance.

Studies have shown that simple physical tests can be reliable indicators for cuff tears [17]. Validated clinical examination tests, such as Jobe's test (empty can test), lag tests, and lift-off tests, have high sensitivity and specificity for detecting the presence of specific rotator cuff muscle tears [18-20]. Additionally, combinations of multiple patient assessment variables can aid in cuff detection and exclusion [21]. However, the reliability of these tests can be dependent on examiner expertise [22], something that would be required in the outpatient setting. These tests may help triage imaging requests and potentially reduce unnecessary scans, especially in those with functional recovery and where conservative management would be a preferable strategy.

It is crucial to select the correct patient for further investigation and management, with comorbidities of aging shown to be an independent factor contributing to poor recovery from surgical intervention [23], indicating that initial assessment may prove appropriate to assess patient goals before further testing. Conservative management has been shown to be non-inferior to surgical intervention, especially in patients aged >50 years and with partial-thickness cuff tears over a one-year period [24], with the difference in outcomes being small. These patients have also been shown to have significantly better outcomes in the initial follow-up period compared to those undergoing surgical procedures, but with a reversal in the trend in the long-term and with significantly improved function in the surgical cohort [25], which may help select patients for further investigations if exhibiting prolonged symptoms. Furthermore, conservative management has been indicated in patients with low functional requirements and moderate symptoms, with close routine monitoring for symptoms relating to tear progression that may necessitate surgical intervention [26].

The limitations of the study must be recognised. The retrospective, single-centre design limits the generalisability of the results to a wider population and introduces institutional practice bias. There will have been an element of selection bias in patient selection for further imaging; clinicians would have opted for further imaging in patients for whom they had more individual concerns, which may have overestimated cuff-tear prevalence and reduced adherence to national guidance. Additionally, using surgery as a clinical endpoint is practical but subjective and reflects more the treatment decisions rather than actual outcome improvement for patients. With this, the absence of patient-reported outcome measures (PROMs) prevents assessment of functional recovery or patient satisfaction following imaging or intervention, which may reduce the validity of our conclusions regarding reducing imaging. Furthermore, the study uses only descriptive analysis with no statistical testing or assessment of the significance of results, which limits causal interpretation and subgroup comparison. Temporal variability will limit results too; pandemic-era service constraints and an evolving local pathway will have affected imaging access and adherence to BESS guidance, and patients with incomplete follow-up will under-represent patients recovering elsewhere and skew outcome rates. Overall, the findings reflect real-world practice patterns and challenges in clinical practices but should be validated prospectively across multiple centres with the inclusion of PROM-based endpoints.

Alteration to guidelines endorsing further imaging in patients aged 40-60 years could create a more cost-efficient process whilst maintaining a more optimal symptom-driven trauma pathway. Consideration of the negative effect of comorbidities on rotator cuff incidence could also help direct clinicians in tear management. Further clinical assessment prior to imaging would enable clinicians to select ideal candidates for potential surgical intervention, considering patient function and quality of life. A high proportion of patients in this study had face-to-face review with imaging to follow, and the use of this method instead of a straight-to-imaging approach would enable functional monitoring and could be more cost-efficient with an unlikely change in outcomes, if considering clinical assessment utility and data comparing outcomes and timeframes of operative versus non-operative management.

## Conclusions

This single-centre study found that very few patients required rotator cuff repair despite radiological evidence of cuff pathology after shoulder dislocation. These results support refining patient selection for imaging in line with BESS recommendations. Most abnormalities identified did not lead to surgery, with physiotherapy achieving excellent outcomes. Further multi-centre prospective studies incorporating PROMs could enhance the validity, generalisability and statistical robustness of these findings. Ongoing routine and indiscriminate imaging may contribute to overtreatment and unnecessary costs, whereas careful clinical assessment can effectively guide decision regarding advanced imaging and management. Overall, this study supports an assessment-driven approach as a more clinically appropriate and resource-efficient alternative to routine advanced imaging for all patients.
